# Covert Communication Scheme for OOK in Asymmetric Noise Systems

**DOI:** 10.3390/s25092948

**Published:** 2025-05-07

**Authors:** Weicheng Xu, Xiaopeng Ji, Ruizhi Zhu

**Affiliations:** School of Electronics and Information Engineering, Nanjing University of Information Science and Technology, Nanjing 210044, China

**Keywords:** covert communication, On–Off Keying, asymmetric noise, Taylor series expansion

## Abstract

Existing covert communication schemes based on On–Off Keying (OOK) have not considered asymmetric noise environments, which limits their applicability in complex communication scenarios such as terahertz and underwater acoustic covert communications. To address this issue, this paper proposes a phase-based OOK coding scheme. In particular, the transmitter Alice can adjust the initial phase of the transmitted symbol to align the signal with the stronger noise components in asymmetric noise communication scenarios, thereby exploiting the masking effect of noise to achieve covert transmission. To quantify performance, the KL divergence and mutual information of the OOK coding scheme are adopted as measures of covertness and transmission performance, respectively. An optimization problem involving the input signal distribution $a_{n}$, signal amplitude β, and initial phase angle θ is formulated and solved to obtain the maximum covert transmission rate. Numerical results demonstrate that in asymmetric noise systems, the initial phase angle and the Gaussian noise components on the real and imaginary axes of the complex plane influence both covertness performance and transmission rate. Adjusting the initial phase towards the direction with lower noise components can maximally suppress noise interference, thereby improving the covertness performance.

## 1. Introduction

The rapid advancement of artificial intelligence (AI) and the Internet of Things (IoT) has led to an increasing demand for efficient and reliable information transmission [1,2,3], which has brought information security issues into focus. Therefore, communication security technologies, such as physical-layer security (PLS) [4,5] and steganography [6,7], have been extensively researched to eliminate the security risks of communication content leakage to eavesdroppers. These technologies can only eliminate the leakage of communication content, but in some fields, such as e-commerce security and unmanned aerospace, their security levels are not high enough to satisfy higher secure communication demands. A new secure communication technology, covert communication, has become one of the critical techniques for security transmission and garnered significant attention across various industries [8,9,10,11,12]. In the covert communication scenario, the transmitter Alice transmits information covertly to the receiver Bob while ensuring that the detector Willie cannot detect their communication behavior [13]. This characteristic is particularly important in scenarios where traditional encryption-based security measures are insufficient.

Therefore, covert communication has attracted significant research interest. Ref. [14] proposed a new method based on bitrate modulation, enabling covert communication among numerous devices over a wide area. Ref. [15] proposes a covert communication method based on Time-Modulated Array (TMA), which outperforms the conventional Phased Array (PA) scheme in terms of both average Covert Age of Information (CAoI) and convergence rate. Ref. [16] investigates a covert communication method based on adaptively selecting between PA and LFDA beamforming schemes, where the transmitter can dynamically choose either PA or LFDA to enhance communication covertness in the presence of spatially random wardens. Meanwhile, covert underwater acoustic communication (CUAC), as an important security method of underwater acoustic communication, has also been extensively explored in recent years [17,18,19]. Ref. [18] proposes an AUV-assisted UACC game (AUACCG) model, which utilizes AUVs to transmit interference signals to weaken the detector’s capability and enhance the security of sensitive data transmission in the Ocean Internet of Things (OIoT). In [19], the authors propose a buoyed-IRS-AUV (BIA) communication method based on intelligent reflecting surfaces (IRSs), which achieves secure and reliable transmission of sensitive data in the Ocean Internet of Things (OIoT) by dynamically adjusting the beam widths and IRS depth. Furthermore, in [20,21,22], covert channels have been proposed to hide communication behavior by using network protocols as carriers, making them elusive. To further enhance security while maintaining transmission performance, covert communication can be integrated with other technologies, such as Binary Phase-Shift Keying (BPSK) [23], Non-Orthogonal Multiple Access (NOMA) [24], and Turbo encoding [25].

In recent years, On–Off Keying (OOK), a modulation technique that employs two symbols {0,A} as input alphabets, has gained widespread application in fields such as terahertz communication and underwater acoustic communication due to its simple structure and high power efficiency. Consequently, investigating covert communication for the OOK coding scheme has become a crucial issue in ensuring information security. However, existing research primarily focuses on symmetric Gaussian noise systems where the noise distribution is uniform [26]. Many practical communication systems face asymmetric noise conditions, which pose new challenges to the codebook’s covertness and transmission performance.

**Figure 1:**
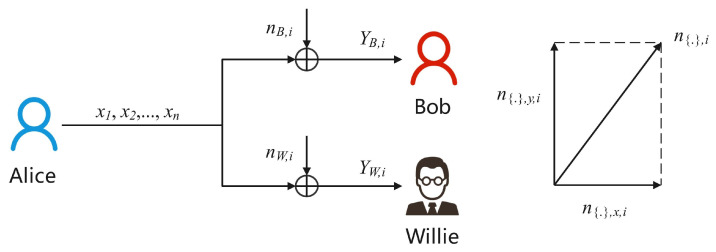
The complex−valued AWGN channel covert communication model.

This paper investigates the covertness performance of the OOK coding scheme over asymmetric noise channels. The communication system model, as shown in Figure 1, includes the legitimate transmitter Alice, the legitimate receiver Bob, and the detector Willie. KL divergence and mutual information are used as the covertness and transmission performance measures, respectively. We explore the impact of signal amplitude and initial phase angles under different noise-component settings on these metrics of the OOK coding scheme. In addition, we explore the scheme that maximizes the transmission rate under the covertness constraint in asymmetric noise systems.

The remainder of this paper is organized as follows: Section 3 presents the covert communication system model over asymmetric noise channels, the OOK codebook construction, Willie’s detection performance analysis, transmission performance analysis, and details the optimization problem. In Section 4, we analyze Willie’s detection ability and the transmission rate between Alice and Bob, and derive the expressions for the OOK coding scheme’s KL divergence and mutual information. In Section 5, we analyze the relationship between optimal amplitude and phase angle according to the covertness constraint and further formulate the corresponding optimization problem. The numerical results are detailed in Section 6, followed by the conclusion presented in Section 7.

## 2. Related Works

We first review the theoretical foundations of covert communication and analyze its performance limits in symmetric Gaussian noise systems. We then summarize the relevant research progress. Finally, we summarize current studies on covert communication in asymmetric noise systems. Our analysis indicates that existing work has largely overlooked the applicability of OOK-based covert communication in asymmetric noise systems, highlighting a significant gap and potential for further investigation.

In existing covert communication research, the limits of achievable performance have been extensively explored in symmetric Gaussian noise channels. In [27], the authors proposed the *square root law* (SRL), which is the theoretical limit of covert communication over Additive White Gaussian Noise (AWGN) channels. Specifically, $O(\sqrt{n})$ bits can be transmitted from Alice to Bob covertly over *n* channels, and as *n* increases to infinity, the transmission rate approaches zero. Additionally, the square-root law applies to binary symmetric channels [28], discrete memoryless channels [29,30,31], multiple-input and multiple-output (MIMO) channels [32,33], multi-user channels [34,35,36], and adversarial channels [37].

Numerous works in covert communication theory focus on improving communication security while maintaining transmission performance. In [24], the authors investigate the performance of covert communication within relay-assisted Non-Orthogonal Multiple Access (NOMA) networks, where the relay acts as a detector, and the transmitter mixes the hidden message with another user’s public message for transmission. In [38], covert-capacity lower bounds and theoretical guarantees for secrecy and reliability are provided for Binary Phase-Shift Keying (BPSK) and root-raised cosine (RRC) pulses in Pulse Amplitude Modulation (PAM). In [39], multiple random-access covert communication protocols over AWGN channels with BPSK modulation achieve low-power random-access covert communication and point-to-point communication.

In most of the aforementioned literature, the research has been conducted in symmetric noise systems. In [26], an OOK coding scheme suitable for complex Gaussian channels is proposed for covert communication. KL divergence and mutual information are calculated to quantify phase-shift gains. However, noise sources include thermal noise, quantization noise, imperfect filters, and environmental wireless signals. In practical applications, due to the constant changes in temperature and environmental noise, noise asymmetry is inevitable. In [40,41], covert communication under noise uncertainty is discussed, and new evaluation methods are proposed to differentiate between bounded and unbounded noise models based on the range of uncertainty. Each model is evaluated to determine the corresponding communication rate under a given covertness requirement. In addition, we notice that the authors in [42] investigated wireless covert communication by employing the BPSK codebook in noise-asymmetric scenarios. The existing research has not fully explored OOK. The simple structure and high power efficiency of OOK have not been fully utilized. Inspired by [26,40,42], this paper studies OOK-based covert communication in asymmetric noise systems, analyzing the impact of signal amplitude and initial phase angles with different noise components on the KL divergence and mutual information of the codebook. It also presents the maximum covert transmission rate achievable under the covertness constraint. Compared to [42], our main contributions are summarized below.

(1) This work investigates the covertness performance of an OOK coding scheme in asymmetric Gaussian noise environments, where the “on” and “off” symbols exhibit asymmetry both in geometric structure and probability distribution. This inherent asymmetry distinguishes OOK codebooks fundamentally from centrally symmetric ones such as BPSK and QAM. As a result, conventional analytical methods that rely on uniformly distributed symbols and symmetric constellations are inadequate for accurately analyzing the covertness and transmission performance of an OOK coding scheme in asymmetric Gaussian noise environments. To address this issue, our work overcomes this limitation by providing a more generalized theoretical framework for analyzing OOK-based covert communication in asymmetric noise systems.
(2) Existing studies have demonstrated that the initial phase angle imposed by the transmitter on the transmitted symbols has a negligible effect on the covertness performance of the codebook in asymmetric noise systems. However, our study reveals that in asymmetric noise systems, the phase angle plays a crucial role in covert communication. Specifically, under covertness constraint, the optimal phase angle that maximizes the transmission rate is not fixed but is influenced by the asymmetry of the noise components, tending to align with the direction of the weaker noise component.

In a nutshell, existing covert communication theories and implementations mainly focus on symmetric noise systems, with limited attention given to asymmetric scenarios. While optimization of initial phase angles and signal amplitudes has demonstrated effectiveness in symmetric channels, the intrinsic asymmetry of OOK codebooks poses a significant challenge [43]. To bridge this gap, our work proposes an OOK-based covert communication scheme specifically designed to achieve covert transmission.

## 3. System Model

### 3.1. Communication Scenario

As shown in Figure 1, we consider a complex-valued AWGN channel covert communication model. This communication model includes the legitimate sender Alice, the legitimate receiver Bob and the detector Willie. Alice needs to covertly send information $X^{n}$ composed of *n* symbols $x={\left\{x_{i}\right\}}_{i=1}^{n}$ to Bob through the asymmetric noise channel while minimizing the possibility of detection by Willie. Meanwhile, Willie tries to detect whether communication behavior is happening between Alice and Bob. When Alice is communicating, every channel uses *i* where i∈{1,2,…n}, and the signals received by Bob and Willie can be respectively expressed as:

(1) $$\begin{array}{cc} Y_{B,i} & =h_{b}x_{i}+n_{B,i}, \end{array}$$

(2) $$\begin{array}{cc} Y_{W,i} & =h_{w}x_{i}+n_{W,i}, \end{array}$$

where $h_{b}$ represents the Alice–Bob channel coefficient, $h_{w}$ represents the Alice–Willie channel coefficient. $n_{B,i}$ and $n_{W,i}$ are independent and identically distributed (i.i.d.) complex-valued Gaussian white noise for the Alice–Bob channel and the Alice–Willie channel, respectively, with zero mean and variances of $2\sigma_{b}^{2}$ and $2\sigma_{w}^{2}$, respectively. The noise satisfies $n_{B,i}∼\mathrm{CN}\left(0,2\sigma_{b}^{2}\right)$, $n_{W,i}∼\mathrm{CN}\left(0,2\sigma_{w}^{2}\right)$. Willie determines whether communication behavior is occurring between Alice and Bob by performing a binary hypothesis test on the received channel output signal $Y_{W}={\left\{Y_{W,i}\right\}}_{i=1}^{n}$.

### 3.2. Codebook Construction

We consider that Alice encodes the message *M* into a codeword of length *n*, denoted as $x^{n}=\left[x_{1},x_{2},\ldots ,x_{n}\right]$, and sends it to Bob. Note that each codeword $x_{i}$ can be uniquely characterized by its amplitude $∥x_{i}∥$ and initial phase $\theta_{x_{i}}$. Therefore, in this paper, we can interchangeably use the notation $\left(∥x_{i}∥,\theta_{x_{i}}\right)$ to represent $x_{i}$. To simplify the subsequent discussion, we represent $\left(∥x_{i}∥,\theta_{x_{i}}\right)$ as $x_{i}$. Next, we will discuss the construction of the OOK codebook with an initial angle in detail.

In the OOK codebook, we use {0,β} as the input alphabet, where each symbol $x_{i}$ can be sent either (0,0) or (β,θ). We denote the input distribution on {(0,0),(β,θ)} as $P_{OOK}\left(x_{i}\right)$. According to the distribution $P_{OOK}^{n}\left(x^{n}\right)=\prod_{i=1}^{n}P_{OOK}\left(x_{i}\right)$, the codeword $x^{n}$ is independently and randomly generated and satisfied:

(3) $$\begin{array}{c} P_{\mathrm{OOK}}\left(x_{i}=\left(\beta ,\theta \right)\right)=1-P_{\mathrm{OOK}}\left(x_{i}=\left(0,0\right)\right)=a_{n}. \end{array}$$

**Figure 2:**
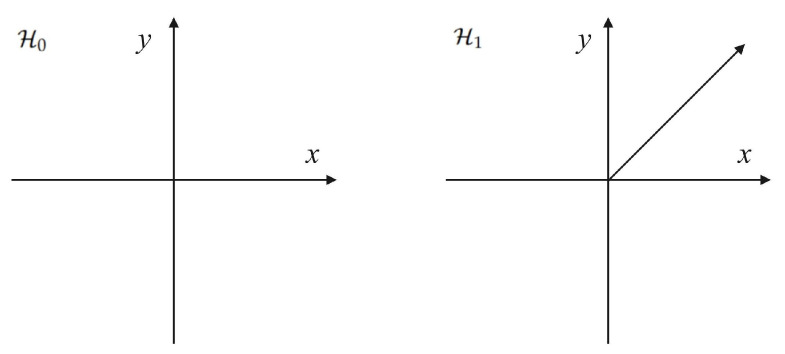
The structure of the OOK codebook.

The structure of the OOK codebook is shown in Figure 2. To intuitively display the structure of the codebook, all non-zero symbols are transmitted with an initial phase angle π/4. When Alice uses the OOK codebook to send messages to Bob, Willie determines whether the communication behavior is happening through a binary hypothesis test.

### 3.3. Hypothesis Test

To determine whether communication is happening between Alice and Bob, Willie conducts signal detection based on the received *n* output signal $Y_{W}={\left\{Y_{W,i}\right\}}_{i=1}^{n}$ and treats it as a binary hypothesis testing problem. The null hypothesis $H_{0}$ indicates that no communication behavior has occurred between Alice and Bob, and the channel contains only Gaussian noise. The alternative hypothesis $H_{1}$ indicates that communication behavior has occurred, and the channel output signal includes both channel noise and the transmitted signal $Y_{W,i}=x_{i}+n_{W,i}$. To detect communication behaviors, Willie attempts to distinguish between the null hypothesis and the alternative hypothesis, which can be described as follows:

(4) $$\begin{array}{cc} \; & H_{0}:Y_{W,i}=n_{W,i}, \end{array}$$

(5) $$\begin{array}{cc} \; & H_{1}:Y_{W,i}=x_{i}+n_{W,i}. \end{array}$$

We define $Q_{0}^{n}$ and $Q_{1}^{n}$ as the probability distributions of the channel output signals received by Willie under the null hypothesis $H_{0}$ and the alternative hypothesis $H_{1}$, respectively. In addition, we define $D_{0}$ as the binary decision when Willie supports $H_{0}$, and $D_{1}$ as the binary decision when Willie supports $H_{0}$. In hypothesis testing, we generally use the total error probability $\xi =P_{FA}+P_{MD}$ as the measure of Willie’s detection performance. $P_{FA}=P\left(D_{1}|H_{0}\right)$ is the Type I error probability (false alarm probability), which means the probability that Willie chooses $D_{1}$ when no communication behaviors take place. $P_{MD}=P\left(D_{0}|H_{1}\right)$ is the Type II error probability (missed detection probability), which means the probability that Willie chooses $D_{0}$ when communication behaviors take place. To ensure achievable results, Alice must guarantee that Willie cannot reliably distinguish whether communication behaviors are taking place, which requires the total error probability to satisfy $P_{FA}+P_{MD}=1$ when making decisions based on the channel output signal. We assume that Willie can obtain the minimum total error probability $\xi^{*}$ with equal prior probability by employing the optimal detection strategy. $\xi^{*}$ can be expressed as:

(6) $$\begin{array}{c} \xi^{*}=1-V\left(Q_{1}^{n}∥Q_{0}^{n}\right). \end{array}$$

Considering the Pinsker’s inequality, the total error probability associated with Willie’s optimal detection strategy is formulated as $\xi^{*}\geq 1-\sqrt{\frac{1}{2}D\left(Q_{1}^{n}∥Q_{0}^{n}\right)}$. $D\left(Q_{1}^{n}∥Q_{0}^{n}\right)$ is the KL divergence between the distributions $Q_{0}^{n}$ and $Q_{1}^{n}$. To achieve effective covert communication, we must ensure that $D\left(Q_{1}^{n}∥Q_{0}^{n}\right)$ is sufficiently small to satisfy:

(7) $$\begin{array}{c} D\left(Q_{1}^{n}∥Q_{0}^{n}\right)\leq \epsilon , \end{array}$$

where ϵ>0 is sufficiently small. Therefore, we choose KL divergence as the covertness performance measure. Under the null hypothesis $H_{0}$, the signal received by Willie is $Y_{W,i}=n_{W,i}$ and the probability distribution of $Y_{W}$ is defined as $Q_{0}^{n}\left(x^{n},y^{n}\right)$ and its expression can be expressed as:

(8) $$\begin{array}{c} H_{0}:Q_{0}^{n}\left(x^{n},y^{n}\right)=\prod_{i=1}^{n}\frac{1}{2\pi \sigma_{w,x}\sigma_{n,y}}\exp\left(-\frac{x_{i}^{2}}{2\sigma_{w,x}^{2}}-\frac{y_{i}^{2}}{2\sigma_{w,y}^{2}}\right), \end{array}$$

where $\sigma_{w,x}^{2}$ and $\sigma_{w,y}^{2}$ represent the variance components of the complex Gaussian noise received by Willie on the real and imaginary axes of the complex plane, satisfying $\sigma_{w,x}^{2}+\sigma_{w,y}^{2}=\sigma_{w}^{2}$. On the other hand, Alice generates codewords (0,0) or (β,θ) with the probabilities described in (3). The probability distribution of $Y_{W}$ under the alternative hypothesis $H_{1}$ can be expressed as:

(9) $$\begin{array}{cc} H_{1}:Q_{1}^{n}\left(x^{n},y^{n}\right)= & \prod_{i=1}^{n}\frac{1}{2\pi \sigma_{w,x}\sigma_{w,y}}[a_{n}\exp\left(-\frac{{\left(x_{i}-\beta \cos\theta \right)}^{2}}{2\sigma_{w,x}^{2}}-\frac{{\left(y_{i}-\beta \sin\theta \right)}^{2}}{2\sigma_{w,y}^{2}}\right)\\ \; & \;+\left(1-a_{n}\right)\exp\left(-\frac{x_{i}^{2}}{2\sigma_{w,x}^{2}}-\frac{y_{i}^{2}}{2\sigma_{w,y}^{2}}\right)]. \end{array}$$

To simplify the representation, we use $D_{0}$ to denote the KL divergence between distributions $Q_{0}^{n}$ and $Q_{1}^{n}$, i.e., $D_{o}≜D\left(Q_{1}^{n}∥Q_{0}^{n}\right)$.

### 3.4. Performance Analysis of the Covert Transmission

In covert communication studies, the transmission rate is commonly measured by the mutual information between Alice and Bob, as it reflects the covert throughput under covertness constraints. Accordingly, we adopt mutual information as the metric for covert transmission rate in this work. The mutual information is defined as $I_{o}\left(x^{n};Y_{B}\right)$ when using the OOK coding scheme. Let $Q_{b,0}$ and $Q_{b,\beta }$ represent the probability distributions of $Y_{B,i}$ when the channel input is (0,0) and (β,θ), respectively; the expressions are given as:

(10) $$\begin{array}{cc} \; & Q_{b,0}=\frac{1}{2\pi \sigma_{b,x}\sigma_{b,y}}\exp\left(-\frac{x^{2}}{2\sigma_{b,x}^{2}}-\frac{y^{2}}{2\sigma_{b,y}^{2}}\right), \end{array}$$

(11) $$\begin{array}{cc} \; & Q_{b,\beta }=\frac{1}{2\pi \sigma_{w,x}\sigma_{w,y}}\exp\left(-\frac{{\left(x-\beta \cos\theta \right)}^{2}}{2\sigma_{b,x}^{2}}-\frac{{\left(y-\beta \sin\theta \right)}^{2}}{2\sigma_{b,y}^{2}}\right). \end{array}$$

The corresponding output distribution of the Alice–Bob channel is denoted as $Q_{b,1}$, and it is defined as:

(12) $$\begin{array}{c} Q_{b,1}≜a_{n}Q_{b,\beta }+\left(1-a_{n}\right)Q_{b,0}. \end{array}$$

In [43], the expression of mutual information for the OOK coding scheme is as follows:

(13) $$\begin{array}{cc} \; & I_{o}\left(x^{n};Y_{B}\right)\\ \; & =n\cdot \left[a_{n}D\left(Q_{b,\beta }∥Q_{b,0}\right)-D\left(Q_{b,1}∥Q_{b,0}\right)\right] \end{array}$$

(14) $$\begin{array}{cc} \; & =n\cdot \left[a_{n}D\left(Q_{b,\beta }∥Q_{b,1}\right)+\left(1-a_{n}\right)D\left(Q_{b,0}∥Q_{b,1}\right)\right] \end{array}$$

### 3.5. Problem Formulation

In this work, we consider the covertness constraint described in Section 3.3. We assume that Willie adopts the optimal detection strategy. We aim to maximize the mutual information in (14) by optimizing the non-zero signal input distribution, signal amplitude, and initial phase angle while satisfying the covertness constraint. The covert communication problem in noise-asymmetric systems can be formulated as:

(15) $$\begin{array}{cc} P1:\max_{a_{n},\beta ,\theta }\; & I\left(x^{n};Y_{B}\right) \end{array}$$

(16) $$\begin{array}{cc} \;s.t.\; & D\left(Q_{0}^{n}∥Q_{1}^{n}\right)\leq \epsilon , \end{array}$$

(17) $$\begin{array}{cc} \; & \beta >0, \end{array}$$

(18) $$\begin{array}{cc} \; & 0\leq \theta <2\pi . \end{array}$$

## 4. Performance Analysis of the Covert Transmission

### 4.1. Analysis of Covertness Performance

As described in the previous section, KL divergence is frequently employed in covert communication as a metric to assess the covertness performance of codebooks. For two probability distributions, $Q_{0}$ and $Q_{1}$, the KL divergence is defined as follows:

(19) $$\begin{array}{c} D\left(Q_{1}∥Q_{0}\right)=\;\int \int \;Q_{1}\left(x,y\right)\log\frac{Q_{1}\left(x,y\right)}{Q_{0}\left(x,y\right)}dxdy. \end{array}$$

Since the channel is memoryless, we have $D\left(Q_{1}^{n}∥Q_{0}^{n}\right)=nD\left(Q_{1}∥Q_{0}\right)$ by the chain rule. Combining the probability distribution $Q_{0}^{n}$ and $Q_{1}^{n}$ described in (8) and (9), the detailed derivation of KL divergence between two single-letter distributions $Q_{1}$ and $Q_{0}$ can be expressed as:

(20) $$\begin{array}{cc} \; & D(Q_{1}∥Q_{0}) \end{array}$$

(21) $$\begin{array}{cc} \; & =\;\int \int \;Q_{1}\left(x,y\right)\log\frac{Q_{1}\left(x,y\right)}{Q_{0}\left(x,y\right)}dxdy\\ \; & =\;\int \int \;Q_{1}\left(x,y\right)\log[a_{n}\exp(-\frac{\left(-2x\beta \cos\theta +\beta^{2}\cos^{2}\theta \right)\sigma_{w,y}^{2}}{2\sigma_{w,x}^{2}\sigma_{w,y}^{2}} \end{array}$$

(22) $$\begin{array}{cc} \; & \;-\frac{\left(-2y\beta \sin\theta +\beta^{2}\sin^{2}\theta \right)\sigma_{w,x}^{2}}{2\sigma_{w,x}^{2}\sigma_{w,y}^{2}})+1-a_{n}]dxdy\\ \; & =\;\int \int \;Q_{1}\left(x,y\right)[\frac{a_{n}\beta \left(y\sin\theta \sigma_{w,x}^{2}+x\cos\theta \sigma_{w,y}^{2}\right)}{\sigma_{w,x}^{2}\sigma_{w,y}^{2}}\\ \; & \;-\beta^{2}(a_{n}\left(\frac{\sigma_{w,x}^{2}\sin^{2}\theta +\sigma_{w,y}^{2}\cos^{2}\theta }{2\sigma_{w,x}^{2}\sigma_{w,y}^{2}}-\frac{{\left(2y\sin\theta \sigma_{w,x}^{2}+2x\cos\theta \sigma_{w,y}^{2}\right)}^{2}}{8\sigma_{w,x}^{4}\sigma_{w,y}^{4}}\right) \end{array}$$

(23) $$\begin{array}{cc} \; & \;+\frac{a_{n}^{2}{\left(2y\sin\theta \sigma_{w,x}^{2}+2x\cos\theta \sigma_{w,y}^{2}\right)}^{2}}{8\sigma_{w,x}^{4}\sigma_{w,y}^{4}})+O\left(\beta^{3}\right)]\;dxdy \end{array}$$

(24) $$\begin{array}{cc} \; & =\;\int \int \;Q_{1}\left(x,y\right)\left(\beta \cdot \mu_{1}-\beta^{2}\cdot \mu_{2}+O\left(\beta^{3}\right)\right)dxdy, \end{array}$$

For clarity of presentation, we define:

(25) $$\begin{array}{cc} \mu_{1} & =\frac{a_{n}\left(y\sin\theta \sigma_{w,x}^{2}+x\cos\theta \sigma_{w,y}^{2}\right)}{\sigma_{w,x}^{2}\sigma_{w,y}^{2}}, \end{array}$$

(26) $$\begin{array}{cc} \mu_{2} & =a_{n}\left(\frac{\sigma_{w,x}^{2}\sin^{2}\theta +\sigma_{w,y}^{2}\cos^{2}\theta }{2\sigma_{w,x}^{2}\sigma_{w,y}^{2}}-\frac{{\left(2y\sin\theta \sigma_{w,x}^{2}+2x\cos\theta \sigma_{w,y}^{2}\right)}^{2}}{8\sigma_{w,x}^{4}\sigma_{w,y}^{4}}\right)\\ \; & \;+\frac{a_{n}^{2}{\left(2y\sin\theta \sigma_{w,x}^{2}+2x\cos\theta \sigma_{w,y}^{2}\right)}^{2}}{8\sigma_{w,x}^{4}\sigma_{w,y}^{4}}. \end{array}$$

In classical covert communication scenarios, the signal amplitude needs to be small enough to achieve covert transmission. Here, we perform a Taylor series expansion on log(·) in (22) to obtain the approximate expression concerning β. To derive the first-order approximation which includes the variables *x*, *y* and their powers, we use the central moments to simplify. Considering a Gaussian probability distribution $D∼N(\mu ,\sigma^{2})$ with mean μ and variance $\sigma^{2}$, the first- and second-order central moments of *x* and *y* are shown as follows:

(27) $$\begin{array}{cc} \; & \;\int \int \;D\cdot xdxdy=\mu , \end{array}$$

(28) $$\begin{array}{cc} \; & \;\int \int \;D\cdot x^{2}dxdy=\mu^{2}+\sigma^{2}. \end{array}$$

According to the distribution $Q_{1}\left(x,y\right)$, we summarize the central moments of *x* and *y*, as well as their second powers, as follows:

(29) $$\begin{array}{cc} x & =a_{n}\cdot \beta \cos\theta +\left(1-a_{n}\right)\cdot 0=a_{n}\beta \cos\theta , \end{array}$$

(30) $$\begin{array}{cc} y & =a_{n}\cdot \beta \sin\theta +\left(1-a_{n}\right)\cdot 0=a_{n}\beta \sin\theta , \end{array}$$

(31) $$\begin{array}{cc} x^{2} & =a_{n}\cdot \left(\beta^{2}\cos^{2}\theta +\sigma_{w,x}^{2}\right)+\left(1-a_{n}\right)\cdot \sigma_{w,x}^{2}=a_{n}\beta^{2}\cos^{2}\theta +\sigma_{w,x}^{2}, \end{array}$$

(32) $$\begin{array}{cc} y^{2} & =a_{n}\cdot \left(\beta^{2}\sin^{2}\theta +\sigma_{w,y}^{2}\right)+\left(1-a_{n}\right)\cdot \sigma_{w,y}^{2}=a_{n}\beta^{2}\sin^{2}\theta +\sigma_{w,y}^{2}. \end{array}$$

The first term of (24) can be obtained as follows:

(33) $$\begin{array}{c} \;\int \int \;Q_{1}\left(x,y\right)\beta \mu_{1}dxdy=\frac{a_{n}^{2}\beta^{2}\left(\sin^{2}\theta \sigma_{w,x}^{2}+\cos^{2}\theta \sigma_{w,y}^{2}\right)}{\sigma_{w,x}^{2}\sigma_{w,y}^{2}}+O\left(\beta^{3}\right). \end{array}$$

Similar to the above process, the second term can be obtained

(34) $$\begin{array}{cc} \; & \;\int \int \;Q_{1}\left(x,y\right)\beta^{2}\mu_{2}dxdy\\ \; & =a_{n}\beta^{2}\frac{\sigma_{w,x}^{2}\sin^{2}\theta +\sigma_{w,y}^{2}\cos^{2}\theta }{2\sigma_{w,x}^{2}\sigma_{w,y}^{2}}+\left(a_{n}^{2}-a_{n}\right)\beta^{2}\frac{y^{2}\sin^{2}\theta \sigma_{w,x}^{4}+x^{2}\cos^{2}\theta \sigma_{w,y}^{4}}{2\sigma_{w,x}^{4}\sigma_{w,y}^{4}} \end{array}$$

(35) $$\begin{array}{cc} \; & \;+\left(a_{n}^{2}-a_{n}\right)\beta^{2}\frac{2\sin\theta \cos\theta \sigma_{w,x}^{2}\sigma_{w,y}^{2}xy}{2\sigma_{w,x}^{4}\sigma_{w,y}^{4}}\\ \; & =\left(a_{n}^{2}-a_{n}\right)\beta^{2}\left[\frac{\left(\sigma_{w,y}^{2}+a_{n}\beta^{2}\sin^{2}\theta \right)\sin^{2}\theta \sigma_{w,x}^{4}+\left(\sigma_{w,x}^{2}+a_{n}\beta^{2}\cos^{2}\theta \right)\cos^{2}\theta \sigma_{w,y}^{4}}{2\sigma_{w,x}^{4}\sigma_{w,y}^{4}}\right] \end{array}$$

(36) $$\begin{array}{cc} \; & \;+\left(a_{n}^{2}-a_{n}\right)\beta^{2}\frac{2a_{n}^{2}\beta^{2}\sin^{2}\theta \cos^{2}\theta \sigma_{w}^{2}}{2\sigma_{w,x}^{4}\sigma_{w,y}^{4}}+a_{n}\beta^{2}\frac{\sigma_{w,x}^{2}\sin^{2}\theta +\sigma_{w,y}^{2}\cos^{2}\theta }{2\sigma_{w,x}^{2}\sigma_{w,y}^{2}} \end{array}$$

(37) $$\begin{array}{cc} \; & =a_{n}\beta^{2}\frac{\sigma_{w,x}^{2}\sin^{2}\theta +\sigma_{w,y}^{2}\cos^{2}\theta }{2\sigma_{w,x}^{2}\sigma_{w,y}^{2}}+\left(a_{n}^{2}-a_{n}\right)\beta^{2}\left(\frac{\sin^{2}\theta \sigma_{w,x}^{2}+\cos^{2}\theta \sigma_{w,y}^{2}}{2\sigma_{w,x}^{2}\sigma_{w,y}^{2}}+O\left(\beta^{4}\right)\right) \end{array}$$

(38) $$\begin{array}{cc} \; & =\frac{a_{n}^{2}\beta^{2}\left(\sin^{2}\theta \sigma_{w,x}^{2}+\cos^{2}\theta \sigma_{w,y}^{2}\right)}{2\sigma_{w,x}^{2}\sigma_{w,y}^{2}}+O\left(\beta^{4}\right). \end{array}$$

Combining (33), (38), and the chain rule, the KL divergence between $Q_{1}^{n}$ and $Q_{2}^{n}$ can be expressed as:

(39) $$\begin{array}{cc} D(Q_{1}^{n}∥Q_{0}^{n}) & =\frac{a_{n}^{2}n\beta^{2}\left(\sin^{2}\theta \sigma_{w,x}^{2}+\cos^{2}\theta \sigma_{w,y}^{2}\right)}{2\sigma_{w,x}^{2}\sigma_{w,y}^{2}} \end{array}$$

(40) $$\begin{array}{cc} \; & =\frac{a_{n}^{2}n\beta^{2}}{2}\left(\frac{\sin^{2}\theta }{\sigma_{w,y}^{2}}+\frac{\cos^{2}\theta }{\sigma_{w,x}^{2}}\right)+O\left(\beta^{4}\right). \end{array}$$

### 4.2. Analysis of Transmission Performance

The expression of mutual information between $x^{n}$ and $Y_{B}$ is as shown in (14). The calculation process for the first term of the mutual information expression is as follows:

(41) $$\begin{array}{cc} \; & a_{n}D\left(Q_{b,\beta }∥Q_{b,1}\right)\\ \; & =-\;\int \int \;\frac{a_{n}}{2\pi \sigma_{b,x}\sigma_{b,y}}\exp\left(-\frac{{\left(x-\beta \cos\theta \right)}^{2}}{2\sigma_{b,x}^{2}}-\frac{{\left(y-\beta \sin\theta \right)}^{2}}{2\sigma_{b,y}^{2}}\right)\\ \; & \;\times \log\left[a_{n}+\left(1-a_{n}\right)\exp\left(-\frac{2x\beta \cos\theta -\beta^{2}\cos^{2}\theta }{2\sigma_{b,x}^{2}}-\frac{2y\beta \sin\theta -\beta^{2}\sin^{2}\theta }{2\sigma_{b,y}^{2}}\right)\right]dxdy. \end{array}$$

We perform Taylor series expansion of the log(·) term in (41) and use central moments for simplification. The approximation of $a_{n}D\left(Q_{b,\beta }∥Q_{b,1}\right)$ is as follows:

(42) $$\begin{array}{cc} \; & a_{n}D\left(Q_{b,\beta }∥Q_{b,1}\right)\\ \; & \approx -a_{n}[\frac{\left(a_{n}-1\right)\beta^{2}\cos^{2}\theta }{2\sigma_{b,x}^{2}}+\frac{\left(a_{n}-1\right)\beta^{2}\sin^{2}\theta }{2\sigma_{b,y}^{2}}\\ \; & \;+\frac{\left(a_{n}-a_{n}^{2}\right)\beta^{2}\cos^{2}\theta }{2\sigma_{b,x}^{2}}+\frac{\left(a_{n}-a_{n}^{2}\right)\beta^{2}\sin^{2}\theta }{2\sigma_{b,y}^{2}}+O\left(\beta^{4}\right)] \end{array}$$

(43) $$\begin{array}{cc} \; & \approx a_{n}\left[\frac{{\left(a_{n}-1\right)}^{2}\beta^{2}\cos^{2}\theta }{2\sigma_{b,x}^{2}}+\frac{{\left(a_{n}-1\right)}^{2}\beta^{2}\sin^{2}\theta }{2\sigma_{b,y}^{2}}+O\left(\beta^{4}\right)\right]. \end{array}$$

Similarly, the calculation of the second term of the mutual information expression is as follows:

(44) $$\begin{array}{cc} \; & \left(1-a_{n}\right)D\left(Q_{b,0}∥Q_{b,1}\right)\\ \; & =\left(a_{n}-1\right)\;\int \int \;\frac{1}{2\pi \sigma_{b,x}\sigma_{b,y}}\exp\left(-\frac{x^{2}}{2\sigma_{b,x}^{2}}-\frac{y^{2}}{2\sigma_{b,y}^{2}}\right)\\ \; & \;\times \log\left[1-a_{n}+a_{n}\exp\left(\frac{2x\beta \cos\theta -\beta^{2}\cos^{2}\theta }{2\sigma_{b,x}^{2}}+\frac{2y\beta \sin\theta -\beta^{2}\sin^{2}\theta }{2\sigma_{b,y}^{2}}\right)\right] \end{array}$$

(45) $$\begin{array}{cc} \; & \approx \left(1-a_{n}\right)\left(\frac{a_{n}^{2}\beta^{2}\cos^{2}\theta }{2\sigma_{b,x}^{2}}+\frac{a_{n}^{2}\beta^{2}\sin^{2}\theta }{2\sigma_{b,y}^{2}}\right). \end{array}$$

Combining Equations (14), (43), and (45) and the chain rule, the mutual information of the OOK coding scheme can be expressed as:

(46) $$\begin{array}{cc} I_{o}\left(x^{n};Y_{B}\right) & \approx \frac{\left(a_{n}-a_{n}^{2}\right)n\beta^{2}\cos^{2}\theta }{2\sigma_{b,x}^{2}}+\frac{\left(a_{n}-a_{n}^{2}\right)n\beta^{2}\sin^{2}\theta }{2\sigma_{b,y}^{2}}+O\left(\beta^{4}\right) \end{array}$$

(47) $$\begin{array}{cc} \; & \approx \frac{\left(a_{n}-a_{n}^{2}\right)\beta^{2}n}{2}\left(\frac{\cos^{2}\theta }{\sigma_{b,x}^{2}}+\frac{\sin^{2}\theta }{\sigma_{b,y}^{2}}\right)+O\left(\beta^{4}\right). \end{array}$$

## 5. Design of Amplitude Gain and Phase Angle

According to the mathematical description of the covertness constraint of problem P1, combined with the KL divergence expression in Section 4.1, we can rewrite the covertness constraint as:

(48) $$\begin{array}{c} \frac{a_{n}^{2}n\beta^{2}}{2}\left(\frac{\sin^{2}\theta }{\sigma_{w,y}^{2}}+\frac{\cos^{2}\theta }{\sigma_{w,x}^{2}}\right)\leq \epsilon . \end{array}$$

The expression of mutual information is derived in Section 4.2. The mutual information within the optimization problem is given by:

(49) $$\begin{array}{c} I\left(x^{n};Y_{B}\right)=\frac{\left(a_{n}-a_{n}^{2}\right)\beta^{2}n}{2}\left(\frac{\cos^{2}\theta }{\sigma_{b,x}^{2}}+\frac{\sin^{2}\theta }{\sigma_{b,y}^{2}}\right). \end{array}$$

To simplify the subsequent derivation process, we construct the following definitions:

(50) $$\begin{array}{cc} \; & M\left(\theta \right)=\frac{\cos^{2}\theta }{\sigma_{b,x}^{2}}+\frac{\sin^{2}\theta }{\sigma_{b,y}^{2}}, \end{array}$$

(51) $$\begin{array}{cc} \; & K\left(\theta \right)=\frac{\sin^{2}\theta }{\sigma_{w,y}^{2}}+\frac{\cos^{2}\theta }{\sigma_{w,x}^{2}}. \end{array}$$

Based on the above definitions and derivations, problem P1 can be further reformulated as problem P2

(52) $$\begin{array}{cc} P2: & \max_{a_{n},\beta ,\theta }\;\frac{\left(a_{n}-a_{n}^{2}\right)\beta^{2}n}{2}M\left(\theta \right) \end{array}$$

(53) $$\begin{array}{cc} \; & \;s.t.\;\frac{a_{n}^{2}n\beta^{2}}{2}K\left(\theta \right)\leq \epsilon , \end{array}$$

(54) $$\begin{array}{cc} \; & \;\;0<\beta , \end{array}$$

(55) $$\begin{array}{cc} \; & \;\;0\leq \theta <2\pi . \end{array}$$

Considering the covertness constraint, we assume that the detector employs an optimal detection strategy. Under this circumstance, the transmitted signal’s maximum amplitude is given by:

(56) $$\begin{array}{c} \beta =\sqrt{\frac{2\epsilon }{a_{n}^{2}nK\left(\theta \right)}}. \end{array}$$

By substituting the maximum amplitude into the mutual information expression of problem P2, problem P2 can be rewritten in the following form:

(57) $$\begin{array}{cc} P3: & \max_{a_{n},\theta }\frac{\epsilon \left(a_{n}-a_{n}^{2}\right)M\left(\theta \right)}{a_{n}^{2}K\left(\theta \right)} \end{array}$$

(58) $$\begin{array}{cc} \; & \;s.t.\;0\leq \theta <2\pi . \end{array}$$

In the optimization process, for the phase-angle range θ from 0 to 2π, there exists an optimal phase angle θ that can maximize mutual information while satisfying the covertness constraint.

## 6. Numerical Results

To evaluate the influence of asymmetric noise systems on the covertness performance of the OOK coding scheme, this section presents detailed simulation analyses to examine the impact of signal amplitude and initial phase angle on both KL divergence and mutual information with diverse noise configurations. Furthermore, we provide simulation results for the maximum covert transmission rate achievable under the covertness constraint in problem P3. Without loss of generality, we set standardized simulation parameters as follows: the channel uses *n* = 10,000, and the Gaussian noise power satisfies $\sigma_{w,x}^{2}+\sigma_{w,y}^{2}=1$, $\sigma_{b,x}^{2}+\sigma_{b,y}^{2}=1$.

**Figure 3:**
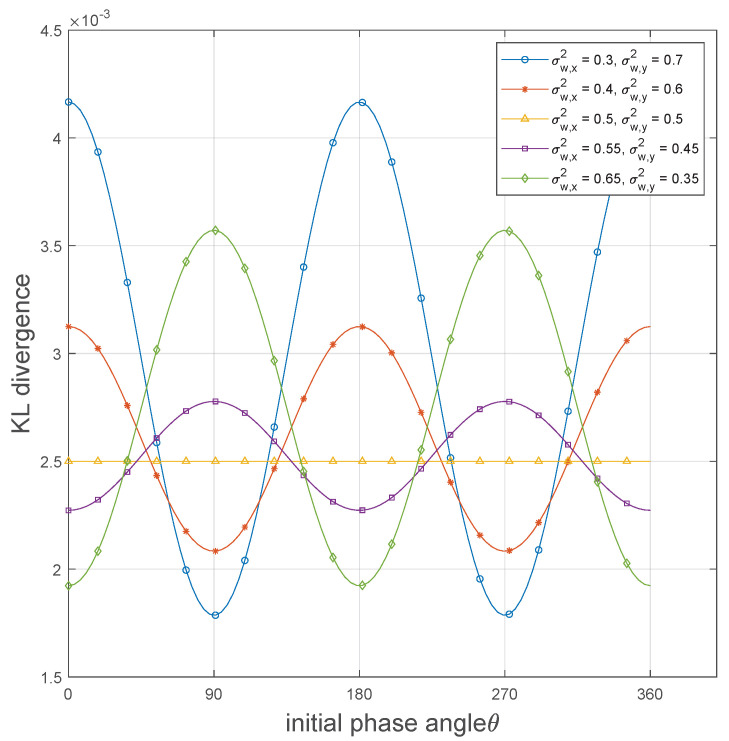
KL divergence versus θ achieved by different channel noise−component configurations.

In Figure 3, we plot the KL divergence of the OOK coding scheme $D(Q_{1}^{n}∥Q_{0}^{n})$ versus the initial phase angle θ with five different parameter configurations, where the noise components on the *x*-axis are 0.3, 0.4, 0.5, 0.55, and 0.65, while the corresponding noise components on the *y*-axis are 0.7, 0.6, 0.5, 0.45, and 0.35. Meanwhile, the channel input distribution parameter $a_{n}$ is set to 0.5, the channel uses *n* = 10,000, and the signal amplitude β is set to 0.001. We can intuitively observe that when the channel noise is symmetric, i.e., $\sigma_{w,x}^{2}=\sigma_{w,y}^{2}$, the KL divergence remains constant, indicating that the choice of phase angle does not affect the covertness performance of the codebook in the symmetric noise channel. However, when the channel noise is asymmetric, the KL divergence exhibits sinusoidal variations as θ changes. It is worth noting that in [26], the phase angle has a minimal impact on covertness performance due to the uniform distribution of noise masking any phase-induced differences in a symmetric noise channel. In contrast, in an asymmetric noise channel, there exists an optimal initial phase angle that can achieve better performance. Figure 3 further reveals the relationship between the optimal phase angle and the channel noise components. Specifically, the optimal phase angle is related to the noise components on the *x*-axis and *y*-axis. When $\sigma_{w,x}^{2}>\sigma_{w,y}^{2}$, the optimal angles are 0 or π, i.e., the signal distributed along the *x*-axis. When $\sigma_{w,x}^{2}<\sigma_{w,y}^{2}$, the optimal angles are $\frac{\pi }{2}$ or $\frac{3\pi }{2}$, i.e., the signal distributed along the *y*-axis. From a physical perspective, transmitting along the axis with higher noise power allows the noise to better obscure the communication signal, thereby improving covertness performance.

**Figure 4:**
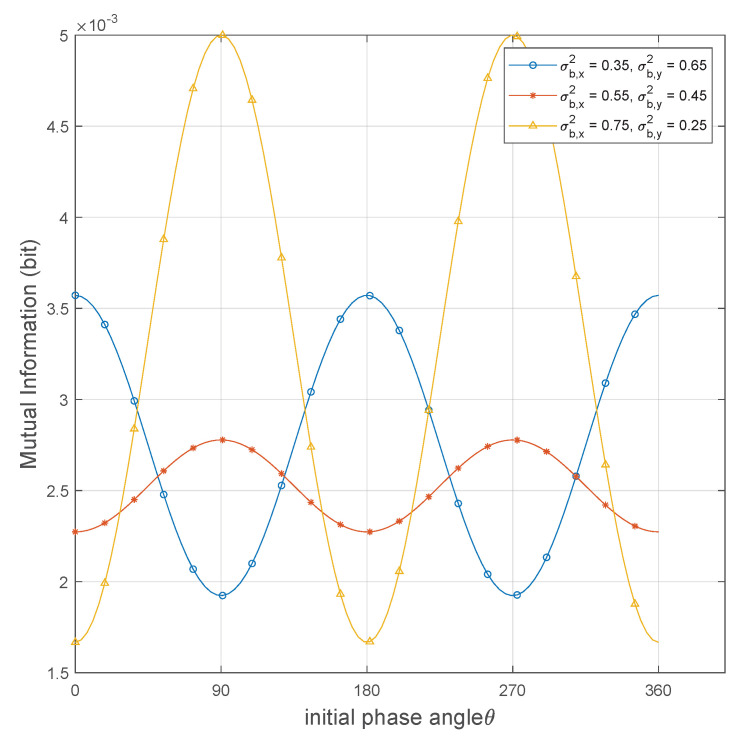
Mutual information versus θ achieved by different channel noise−component configurations.

In Figure 4, we plot the mutual information of the OOK coding scheme versus the initial phase angle θ with three different channel noise-component configurations, where noise components on the *x*-axis are 0.35, 0.55, and 0.75, while the corresponding noise components on the *y*-axis are 0.65, 0.45, and 0.25. It is intuitively clear from the figure that the mutual information of the codebook exhibits a sinusoidal fluctuation pattern with variations in θ. This trend shows a significant similarity to the KL divergence versus phase angle, as shown in Figure 3, indicating that the initial phase angle influences not only the KL divergence but also the mutual information of the codebook. The selection of the optimal phase angle is determined by the $\sigma_{b,x}^{2}$ and $\sigma_{b,y}^{2}$.

**Figure 5:**
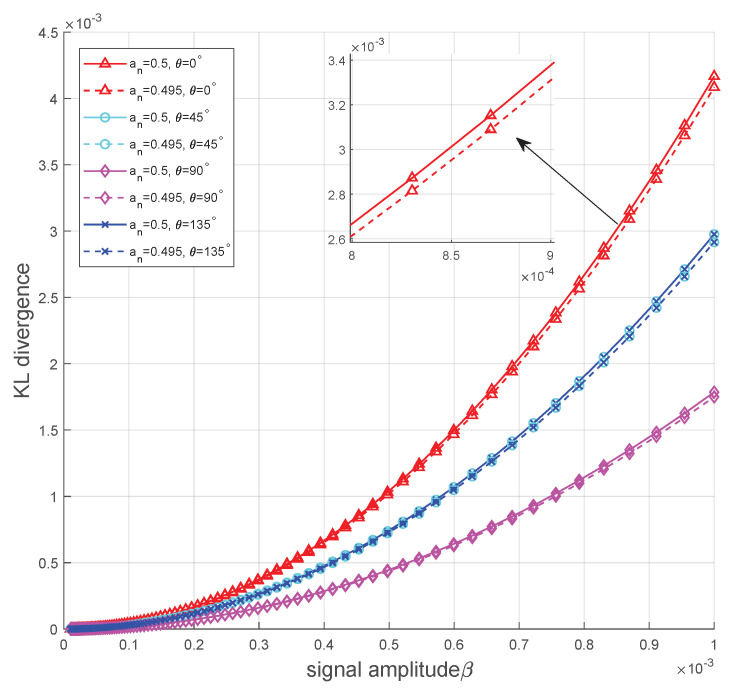
KL divergence versus the signal amplitude β with $\sigma_{w,x}^{2}=0.3$ and $\sigma_{w,y}^{2}=0.7$.

Figure 5 depicts the KL divergence of the OOK coding scheme versus signal amplitudes β with four phase-angle configurations. To examine the impact of the initial phase angle θ on covertness performance, θ is set to 0, $\frac{\pi }{4}$, $\frac{\pi }{2}$ and $\frac{3\pi }{4}$, respectively. It can be observed from the figure that as the signal amplitude increases, the KL divergence also rises, which means an increase in signal amplitude results in poorer performance. Furthermore, the value of the initial phase angle significantly affects the KL divergence. When θ=0, the KL divergence reaches its maximum. As θ increases to $\frac{\pi }{2}$, the KL divergence decreases and attains its minimum. Beyond $\frac{\pi }{2}$, as θ continues to increase toward $\frac{3\pi }{4}$ and further to π, the KL divergence rises again. This trend is consistent with the observations in Figure 3, further validating that the optimal initial phase angle exhibits a strong correlation with the channel noise component on the *x*-axis and *y*-axis. In particular, when the noise component on the *x*-axis is smaller, the optimal angles are $\frac{\pi }{2}$ or $\frac{3\pi }{2}$. In this case, as θ increases from 0 to $\frac{\pi }{2}$ or from π to $\frac{3\pi }{2}$, the noise-masking effect on the signal improves and the KL divergence gradually reaches its minimum. Conversely, when the noise component on the *x*-axis is larger, the optimal angles are 0 or π. As θ increases from 0 to $\frac{\pi }{2}$ or from π to $\frac{3\pi }{2}$, the noise-masking effect on the signal weakens, causing the KL divergence to reach its maximum. Additionally, Figure 5 reveals the impact of channel input distribution $a_{n}$ on the KL divergence. The figure presents the KL divergence corresponding to different $a_{n}$ under the same θ. As $a_{n}$ decreases, the KL divergence also decreases, indicating that a sparser codeword distribution can effectively improve covertness performance.

**Figure 6:**
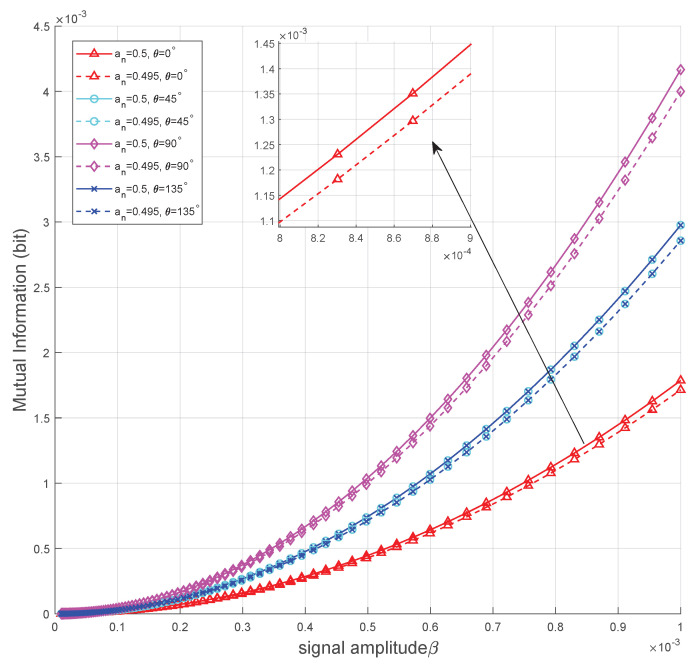
Mutual information versus signal amplitudes with $\sigma_{w,x}^{2}=0.7$ and $\sigma_{w,y}^{2}=0.3$.

In Figure 6, we plot the mutual information of the OOK coding scheme versus signal amplitudes β with four phase configurations, where the phase angle θ is set to 0, $\frac{\pi }{4}$, $\frac{\pi }{2}$ and $\frac{3\pi }{4}$, respectively. The remaining parameter settings are consistent with those in Figure 5. The figure shows that as the signal amplitude increases, the mutual information also rises, following a trend similar to that of KL divergence versus β. However, the effect of the θ on mutual information differs from its influence on KL divergence. Specifically, when the noise component on the *x*-axis is larger, the optimal angles are chosen from $\frac{\pi }{2}$ or $\frac{3\pi }{2}$. As θ increases from 0 to $\frac{\pi }{2}$ or from π to $\frac{3\pi }{2}$, the interference caused by noise in information transmission is reduced, and mutual information gradually reaches its maximum. Conversely, when the noise component on the *x*-axis is larger, the trend is reversed.

**Figure 7:**
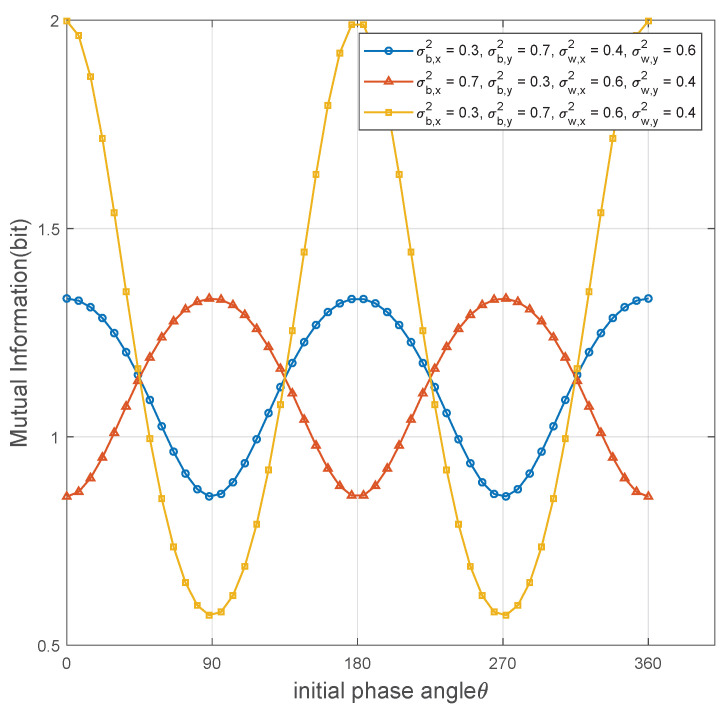
Mutual information versus initial phase angle θ achieved by the OOK coding scheme.

Figure 7 shows the mutual information versus the initial phase angle θ achieved by the OOK coding scheme under covertness constraint. We consider three different communication scenarios: in Scenario 1, $\sigma_{b,x}^{2}>\sigma_{b,y}^{2},\sigma_{w,x}^{2}>\sigma_{w,y}^{2}$; in Scenario 2, $\sigma_{b,x}^{2}<\sigma_{b,y}^{2},\sigma_{w,x}^{2}<\sigma_{w,y}^{2}$; in Scenario 3, $\sigma_{b,x}^{2}<\sigma_{b,y}^{2},\sigma_{w,x}^{2}>\sigma_{w,y}^{2}$. Each scenario represents different channel noise distributions. We can observe that the transmission rate trend is directly influenced by the distribution of the noise components on the Alice-Bob channel. Similar to the trend shown in Figure 6, the direction with larger channel noise components has a more significant inhibitory effect on signal transmission. When θ is closer to the direction with larger noise components, the noise interference on the signal increases, leading to a decrease in mutual information and thus reducing the transmission rate. In addition, the figure reveals that the value of transmission rate is jointly determined by the noise distribution on Alice-Bob and Willie-Willie channels in asymmetric noise systems.

**Figure 8:**
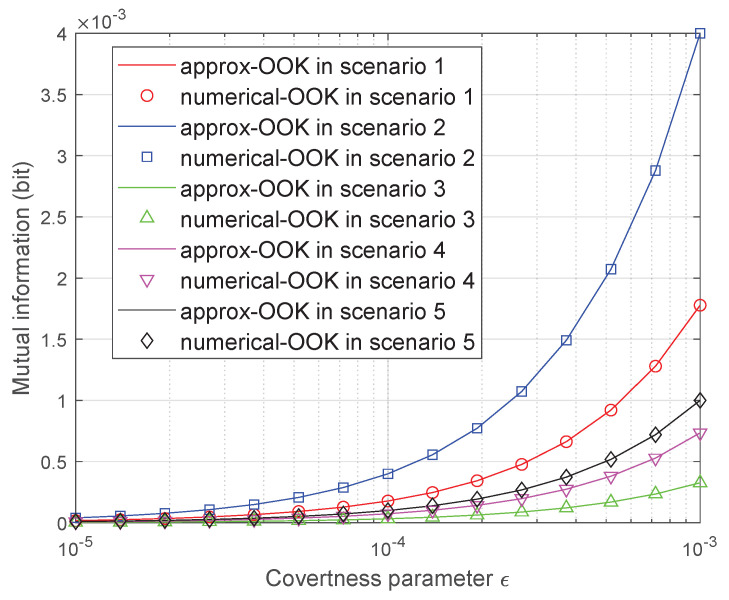
Mutual information versus ϵ achieved by the OOK coding scheme.

In Figure 8, we plot $I(x^{n};Y_{B})$ of the OOK coding scheme versus the covertness parameters ϵ for five different scenarios and provide approximation results. In Scenario 1, $\sigma_{b,x}^{2}=0.3$, $\sigma_{b,y}^{2}=0.7$, $\sigma_{w,x}^{2}=0.4$, $\sigma_{w,y}^{2}=0.6$; in Scenario 2, $\sigma_{b,x}^{2}=0.3$, $\sigma_{b,y}^{2}=0.7$, $\sigma_{w,x}^{2}=0.6$, $\sigma_{w,y}^{2}=0.4$; in Scenario 3, $\sigma_{b,x}^{2}=0.7$, $\sigma_{b,y}^{2}=0.3$, $\sigma_{w,x}^{2}=0.4$, $\sigma_{w,y}^{2}=0.6$; in Scenario 4, $\sigma_{b,x}^{2}=0.7$, $\sigma_{b,y}^{2}=0.3$, $\sigma_{w,x}^{2}=0.6$, $\sigma_{w,y}^{2}=0.4$; in Scenario 5, $\sigma_{b,x}^{2}=\sigma_{b,y}^{2}=\sigma_{w,x}^{2}=\sigma_{w,y}^{2}=0.5$. The initial phase is set to 0. When the direction of the lower noise-power component in the Alice–Bob channel aligns with the initial phase of the transmitted codeword, the signal undergoes less attenuation along that direction, enhancing the achievable mutual information. Consequently, as observed in Figure 8, the transmission rate in Scenario 1 and Scenario 2 is significantly higher than that in the case of symmetric distributed noise power, as represented by Scenario 5. Furthermore, if the initial phase were instead set to π/2 under the same channel conditions, the signal energy would primarily align with the direction of higher noise power, resulting in stronger interference and a notable reduction in the maximum achievable covert transmission rate. Moreover, our approximation closely aligns with the outcomes derived from exact numerical results, thereby validating our theory.

## 7. Conclusions

This paper investigates the covertness performance of OOK coding scheme in asymmetric noise systems and proposes a covert communication coding scheme based on the initial phase angle. The proposed scheme improves covertness by aligning the optimal phase shifts of transmitted symbols with the direction of minimum noise power, effectively utilizing channel noise to mask transmissions and minimize their detectability. We derive the closed-form expressions of OOK coding scheme’s KL divergence and mutual information in asymmetric noise systems, where KL divergence serves as the covertness measure and mutual information as the transmission performance measure. We formulate an optimization problem that maximizes the transmission rate under covertness constraints and solves it. Moreover, we propose an optimal covert transmission strategy that aligns the transmitted codewords’ initial phase angle with the direction of lower noise components, effectively mitigating noise-induced interference. Numerical results demonstrate that in asymmetric noise systems, the initial phase angle significantly influences both KL divergence and the transmission rate. Furthermore, the optimal initial phase angle is significantly influenced by the noise-component magnitude, and it is effective in reducing noise interference by adjusting it toward the direction with lower noise components.

## Data Availability

Data are contained within the article.

## Abbreviations

The following abbreviations are used in this manuscript:

BPSK	Binary Phase-Shift Keying
PLS	Physical-Layer Security
NOMA	Non-Orthogonal Multiple Access
AWGN	Additive White Gaussian Noise
KL	Kullback–Leibler divergence
IoT	Internet of Things
RRC	root-raised cosine
